# Reduction of Methylglyoxal-Induced Glycation by Pyridoxamine Improves Adipose Tissue Microvascular Lesions

**DOI:** 10.1155/2013/690650

**Published:** 2013-04-07

**Authors:** Tiago Rodrigues, Paulo Matafome, Daniela Santos-Silva, Cristina Sena, Raquel Seiça

**Affiliations:** ^1^Laboratory of Physiology, Institute of Biomedical Research on Light and Image (IBILI), Faculty of Medicine, University of Coimbra, 3000-354 Coimbra, Portugal; ^2^Center of Ophthalmology, IBILI, Faculty of Medicine, University of Coimbra, 3000-354 Coimbra, Portugal

## Abstract

*Background and Aims*. Adipose tissue dysfunction results from many factors, including glycation-induced microvascular damages. We tested the usefulness of inhibiting methylglyoxal-induced glycation to adipose tissue microvasculature in this work, using the antioxidant and dicarbonyl scavenger drug pyridoxamine. *Methods*. A group of Wistar rats was treated daily with methylglyoxal (MG, 75 mg/Kg/day, 8 weeks). Half of this group was treated with pyridoxamine in the following 4 weeks (Pyr) (100 mg/Kg/day) and the other half did not have any further treatment (MG). A group of Wistar rats without MG treatment was used as control (C). *Results*. MG group showed decreased HDL cholesterol and increased plasma free fatty acids levels, what was reverted by pyridoxamine. MG also caused an increase of tissue CEL levels (glycation marker), as well as increased staining of PAS and Masson Trichrome-positive components. Pyridoxamine led to CEL and TGF-**β** levels similar to those observed in control rats and inhibited the accumulation of PAS and Masson Trichrome-positive components. MG caused a decrease of Bcl-2/Bax ratio (marker of apoptosis) and vWF staining (microvascular marker), what was partially reverted by the treatment with pyridoxamine. *Conclusions*. Preventing methylglyoxal-induced accumulation of glycated and fibrotic materials using pyridoxamine improves the microvascular lesions of the adipose tissue.

## 1. Introduction

Adipose tissue dysfunction relies on many and heterogeneous factors, including impaired secretory function, insulin resistance, and lipolysis, which are believed to be caused by the activation of inflammatory mechanisms. In turn, inflammation is activated as a consequence of excessive lipid uptake, adipocyte hypertrophy, and hypoxia. Besides the limited ability of oxygen to diffuse between hypertrophic adipocytes, hypoxia also results from microvascular dysfunction and decreased compensatory angiogenesis [1–4]. These mechanisms are thought to contribute to the development and progression of type 2 diabetes. Recently, our group demonstrated that microvascular dysfunction and concomitant hypoxia may be caused by methylglyoxal-induced glycation, what may constitute a new factor for adipose tissue dysfunction during type 2 diabetes progression [1]. Thus, new strategies improving microvascular function of adipose tissue may strongly contribute to prevent these mechanisms.

Methylglyoxal (MG) is a highly reactive dicarbonyl compound, endogenously formed during lipid peroxidation and glycolysis. Irreversible formation of advanced glycation end-products (AGE) from methylglyoxal is one of the pathways responsible for diabetes-associated complications, not only due to increased aminoacid crosslinks and protein misfolding, but also due to the RAGE activation (AGE receptor) [2, 3]. We showed that increased methylglyoxal-induced AGEs accumulation in adipose tissue of Wistar rats leads to hypoadiponectinemia, increased plasma free fatty acids, fibrosis, decreased irrigation, hypoxia, higher levels of apoptotic markers (ratio Bcl-2/Bax and caspase 3), and macrophage recruitment [1]. As well, other authors showed that MG-induced AGE accumulation leads to microvascular dysfunction in other models [5–8].

Pyridoxamine is a vitamin B_6_ derivative, which belongs to the B vitamins family, a group of vitamins known as coenzymes of several metabolic cellular pathways. Pyridoxamine is known to be an inhibitor of the Maillard reaction and a blocker of AGE formation from Amadori products, mainly because it acts as a dual scavenger of free radical and carbonyl species. Pyridoxamine was observed to decrease AGE accumulation, in part due to increased glyoxalase-I expression and decreased oxidative stress and RAGE levels in several models of diabetic complications [6, 9–12].

This work was designed to assess the usefulness of AGE inhibition in the adipose microvasculature, after methylglyoxal-induced glycation. Pyridoxamine was selected due to its double inhibitory functions over AGE formation.

## 2. Materials and Methods

### 2.1. Reagents

Unless otherwise stated all reagents were purchased to Merck Darmstadt (Germany), Sigma-Aldrich (EUA) or Pancreac Química SA (Spain). Antibodies used were directed to actin (MAB1501R, Millipore, USA), CEL (KH025, TransGenic Inc., Japan), RAGE (ab3611, Abcam, UK), TGF-*β* (MAB240, R&D Systems Inc.), Bax, Bcl-2 (SC 6236 and SC7382, Santa Cruz Biotechnology, USA), and vWF (A0082, DAKO, USA).

### 2.2. Animal Models

In this work we studied 6-month-old Wistar rats, from our breeding colonies at the Faculty of Medicine, University of Coimbra. A group of Wistar rats was treated with methylglyoxal in the daily water (Sigma, USA) during 8 weeks (75 mg/Kg/day, diluted in the water). After this period, these rats were divided in two subgroups: one was treated with Pyridoxamine (Pyr) (Sigma, USA) during the following 4 weeks (100 mg/Kg/day diluted in the daily water) [13, 14], whereas the other did not have any kind of treatment (MG). The control group (C) did not have any treatment during the whole period of the experiment (*n* = 6 per group). MG administration was suspended at the moment of pyridoxamine treatment initiation, in order to avoid *ex vivo* interactions with pyridoxamine. Animals were kept (2 per cage) under standard ventilation, temperature (22–24°C), humidity (50–60%), and light (12 hours light/12 hours darkness) with free access to water and food (standard diet AO4, Panlab, Barcelona). In treated animals, water consumption was monitored daily for dosage adjustment purposes. The experimental protocol was approved by the local Institutional Animal Care and Use Committee, and all the procedures were performed by licensed users (FELASA).

### 2.3. *In Vivo *Analysis and Sample Collection

At the end of the treatment, body weight was determined. Glycemia, fasting (16 hours) and 2 hours after intraperitoneal glucose administration (1.8 g/Kg), was measured in the tail vein through the glucose oxidase method, using a glucometer and reactive test stripes (Elite-Bayer SA, Portugal). Glycated hemoglobin was determined using the DCA2000+ system (Siemens, Portugal).

Blood samples were obtained through cardiac puncture from anesthetized animals (ketamine chloride (75 mg/kg, i.m., Parke-Davis, Ann Arbor, USA) and chlorpromazine chloride (2.65 mg/kg, im, Lab. Vitória, Portugal)), after 16 hours fasting. Serum and plasma were collected using tubes BD Vacutainer and BD Vacutainer K3E, with 5.4 mg EDTA (UK), respectively. Blood was centrifuged at 3500 ×g, 4°C, 10 minutes and plasma and serum were aliquoted and stored at −80°C. After blood collection, animals were sacrificed and epididymal adipose tissue was harvested, washed in 0.9% NaCl, and immediately stored in 10% formalin or frozen at −80°C.

Serum levels of cholesterol (total and HDL) and triglycerides were determined using commercial kits (Olympus-Diagnóstica, Portugal, Produtos de Diagnóstico SA, Portugal). Serum concentration of adiponectin was determined by commercially available ELISA kits (Adiponectin immunoassay kit KRP0041, Invitrogen). Plasma levels of free fatty acids were assessed spectrophotometrically using the half-micro test (11383175001 Roche Diagnostic, Germany).

### 2.4. Western Blotting

Tissue sections of 300 mg were homogenized in a buffer containing 25 mM Tris, 150 mM NaCl, 1% Triton X-100, 1 mM EDTA, 1 mM EGTA, 10 mM PMSF, and 40 *μ*L/g tissue of proteases inhibitor cocktail (Sigma, USA), pH = 7.7 and centrifuged at 14000 ×g, 20 minutes, 4°C. Supernatants were collected, centrifuged again, and protein concentration was determined using the BCA method (Pierce, USA). Samples (50 *μ*g) were separated by SDS-PAGE in 10% acrylamide gels and transferred to PVDF membranes. Membranes were blocked with TBST solution (25 mM Tris-HCl, 150 mM NaCl, 0.1% Tween, pH = 7.6) supplemented with 5% BSA. Membranes were then incubated overnight at 4°C with the respective primary antibodies (CEL, RAGE, TGF-*β*, Bcl-2 and Bax, diluted 1 : 1000 in TBST solution supplemented with 1% BSA) and during 2 hours at room temperature with the secondary antibodies (anti-rabbit/mouse 1 : 10000; GE Healthcare, UK). Membranes were incubated with ECF and revealed using the Typhoon system (GE Healthcare Life Sciences, UK). Membrane analysis was performed using the software Image Quant (Molecular Dynamics, USA). 

### 2.5. Histology

Tissue sections (4 *μ*m) were stained with Periodic Acid Schiff (PAS; carbohydrates and glycoconjugates) and Masson Trichrome (collagen/connective tissue), two slices per animal, three animals per group. Immune staining was performed after paraffin removal, hydration, and blocking, following the recommendation of the manufacturer (DAB150 immunoperoxidase secondary detection system JH1743622, Millipore, USA). Sections were incubated overnight at 4°C with the primary antibody (TGF-*β* and vWF, diluted 1 : 100 in PBS) and during 1 hour at room temperature with the secondary antibodies (Alexa Fluor 488 goat anti-rabbit IgG and Alexa Fluor 568 goat anti-mouse IgG, Invitrogen, USA). Finally, sections were mounted with mounting medium (DAKO, USA) and analyzed in a fluorescent microscope (Leica DMIRE2, Leica Microsystems, Germany). Fluorescence was adjusted to avoid background fluorescence of paraffin. For vWF quantification ten images were captured randomly (images containing large vessels were excluded) from two slices per animal/three animals per group. Fluorescence quantification was performed using the software ImageJ (NIH, USA).

### 2.6. Statistical Analysis

Results are presented as mean ± SEM. One-way ANOVA test was used to determine statistical differences. Post hoc Tukey test was performed to assess the differences between groups. *P* < 0.05 was considered significant.

## 3. Results

### 3.1. Metabolic Profile and Functional Systemic Markers

**Figure 1:**
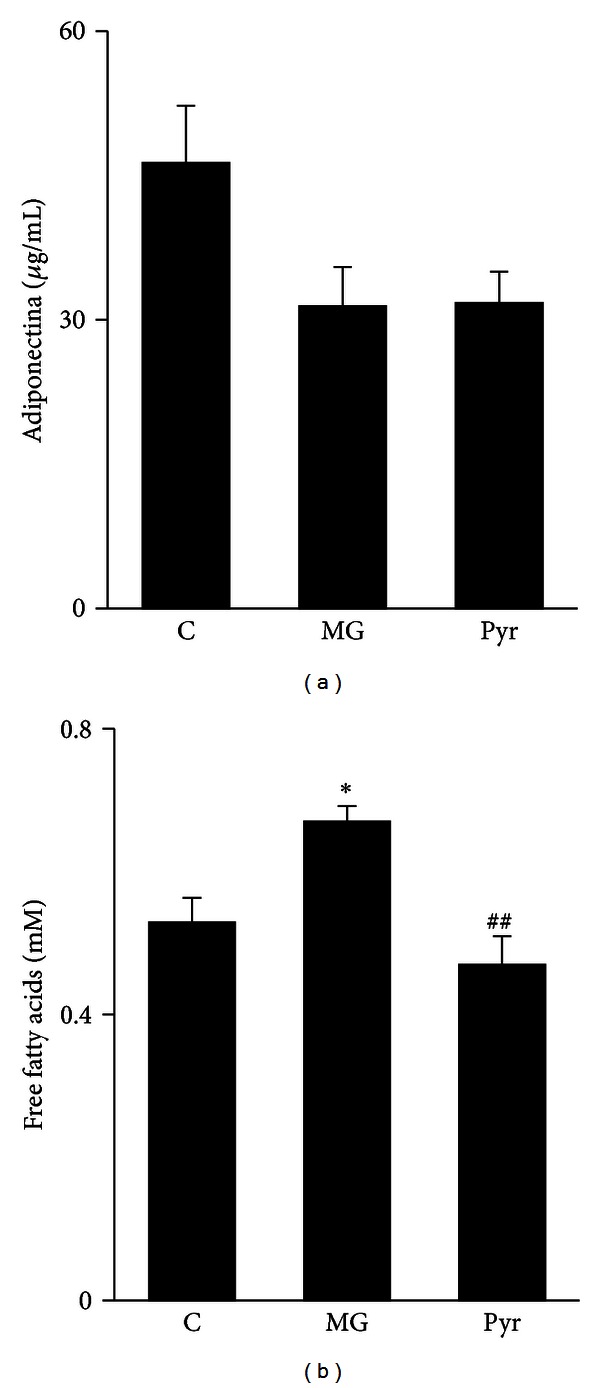
Serum adiponectin (*μ*g/mL) (a) and plasma free fatty acids levels (mM) (b). C: Control Wistar rats; MG: Wistar rats with methylglyoxal administration; Pyr: Wistar rats with methylglyoxal administration and pyridoxamine treatment. *n* = 6 in each group. *Different from C. ^#^Different from MG. 1 symbol *P* < 0.05; 2 symbols *P* < 0.01.

MG administration during 8 weeks (MG group), as well as the treatment with pyridoxamine during 4 weeks (Pyr group), did not result in significant alterations of body weight, glycemia, HbA1c, triglycerides, and total cholesterol, compared to the control (C) group (Table 1). However, MG group showed a significant decrease of HDL cholesterol levels, compared to the C group (*P* < 0.05), which was reverted by pyridoxamine treatment (Pyr group) (*P* < 0.05) (Table 1). Regarding serum adiponectin levels, no significant alterations were found (Figure 1(a)). On the other hand, MG administration led to increased circulating free fatty acids levels, when compared to C group (*P* < 0.05), suggesting adipocyte dysfunction. In turn, the treatment with pyridoxamine normalized plasma free fatty acids levels (*P* < 0.01) (Figure 1(b)).

### 3.2. Tissue Markers of Microvascular Dysfunction

#### 3.2.1. Glycation

**Figure 2:**
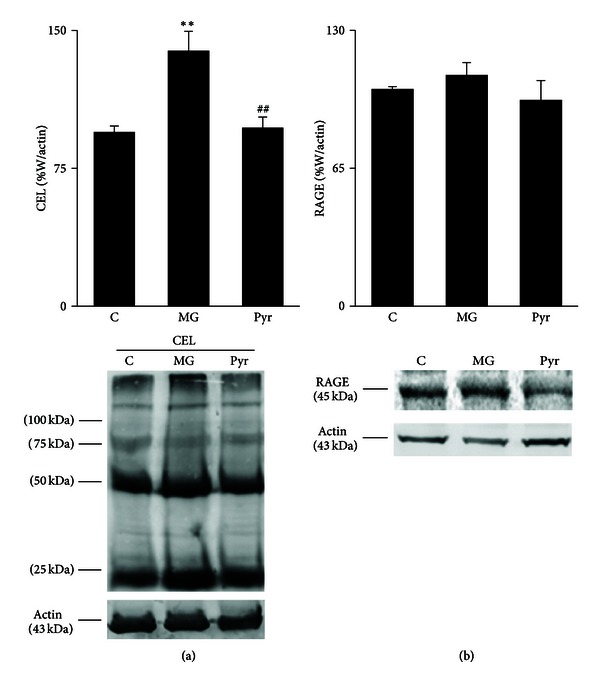
Adipose tissue levels of CEL (a) and RAGE (b), calculated as percentage of C. Representative Western blots of CEL and RAGE are shown. C: Control Wistar rats; MG: Wistar rats with methylglyoxal administration; Pyr: Wistar rats with methylglyoxal administration and pyridoxamine treatment. *n* = 6 in each group. *Different from C. ^#^Different from MG. 2 symbols *P* < 0.01.

**Figure 3:**
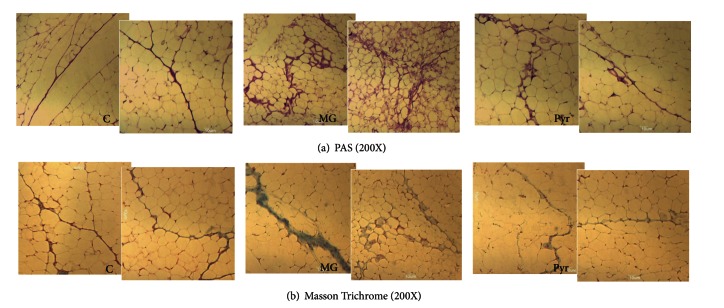
Histological analysis of adipose tissue show PAS staining (200X) (a) and Masson Trichrome staining (200X) (b). C: Control Wistar rats; MG: Wistar rats with methylglyoxal administration; Pyr: Wistar rats with methylglyoxal administration and pyridoxamine treatment.

Glycation is known to be related to microvascular dysfunction. MG treatment during 8 weeks resulted in increased N-epsilon-(carboxyethyl)lysine (CEL) accumulation in adipose tissue, compared to the C group (*P* < 0.01) (Figure 2(a)), but did not cause significant alterations of RAGE expression (Figure 2(b)). Pyridoxamine administration significantly reverted CEL accumulation in adipose tissue (*P* < 0.01), leading to values similar to control rats (Figure 2(a)). PAS staining represents the accumulation of carbohydrates and nonspecific glycated materials. In accordance with CEL levels, a substantial increase of PAS material was observed in the adipose tissue of MG-treated rats, when compared to the C group. This accumulation was reverted by pyridoxamine treatment, leading to a phenotype similar to control rats (Figure 3(a)). 

#### 3.2.2. Fibrosis

**Figure 4:**
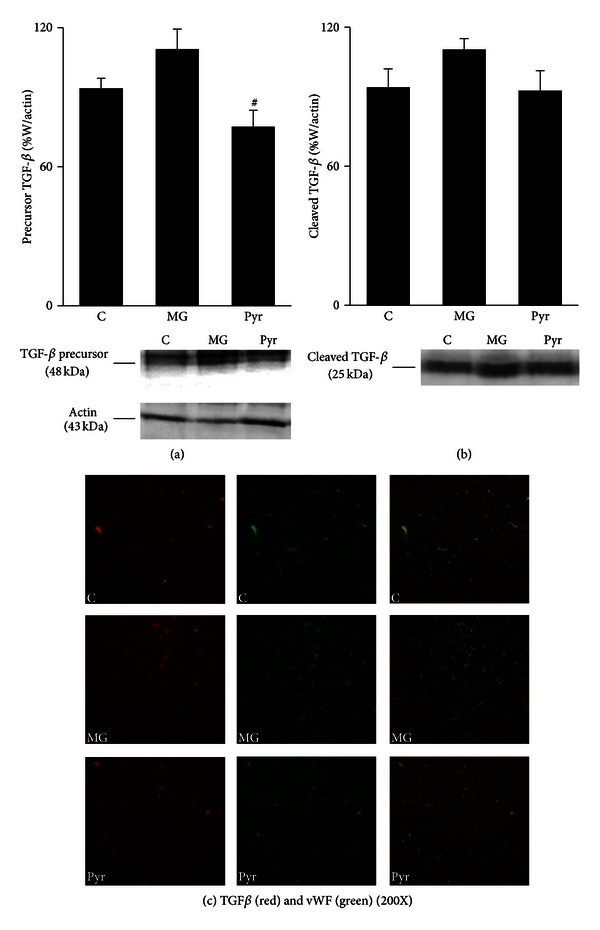
Adipose tissue levels of precursor (a) and cleaved (b) forms of TGF-*β*, calculated as percentage of W. Representative Western blots of TGF-*β* forms are shown. Immunohistochemistry images of adipose tissue show TGF-*β* (red), vWF (green), and merge staining (200X) (c). C: Control Wistar rats; MG: Wistar rats with methylglyoxal administration; Pyr: Wistar rats with methylglyoxal administration and pyridoxamine treatment. *n* = 6 in each group. ^#^Different from MG. 1 symbol *P* < 0.05.

Masson Trichrome staining shows components rich in collagen. The administration of MG during 8 weeks resulted in more fibrotic components in the adipose tissue. More, the staining was observed in PAS-positive regions. Once again, the treatment with pyridoxamine resulted in decreased fibrotic material, resulting in a phenotype similar to control rats (Figure 3(b)). As well, the transforming growth factor-*β* (TGF-*β*) precursor was observed to be decreased after pyridoxamine treatment, when compared to the MG group (*P* < 0.05) (Figure 4(a)). No significant differences were observed in the cleaved form of the TGF-*β* (Figure 4(b)). The histological staining of TGF-*β* showed accumulation in the capillaries, as suggested by its colocalization with the Von Willebrand factor (Figure 4(c)).

#### 3.2.3. Apoptosis

**Figure 5:**
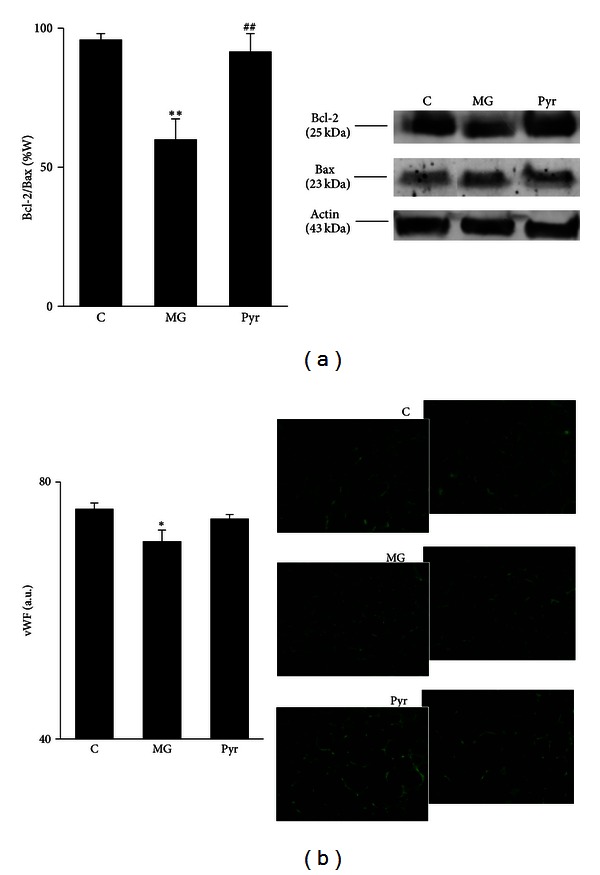
Bcl-2/Bax ratio in adipose tissue: (a) representative Western blots of Bcl-2 and Bax are shown. Immunohistochemistry images show vWF staining, and the respective quantification of fluorescence (b). C: Control Wistar rats; MG: Wistar rats with methylglyoxal administration; Pyr: Wistar rats with methylglyoxal administration and pyridoxamine treatment. *n* = 6 in each group. *Different from C. ^#^Different from MG. 1 symbol *P* < 0.05; 2 symbols *P* < 0.01.

The ratio between Bcl-2 and Bax can be used as an indicator of the predisposition for cellular death by apoptosis. The decrease of this ratio in the MG group, when compared to the C group (*P* < 0.01), indicates that MG-induced glycation leads to a cellular death predisposition. These effects were completely reverted by the treatment with pyridoxamine (*P* < 0.01) (Figure 5(a)). 

#### 3.2.4. Endothelial Dysfunction

Quantification of vWF fluorescent staining was used as a marker of adipose tissue microvasculature, as it is a marker of endothelial cells and is present in the circulation. MG administration resulted in a significant decrease of adipose tissue vWF levels, when compared to control rats (*P* < 0.05). After pyridoxamine treatment a significant difference in relation to the C group was no longer observed, despite that no significant differences were observed between MG and Pyr groups (Figure 5(b)).

## 4. Discussion

This study investigates the scavenger properties and beneficial actions of pyridoxamine in methylglyoxal-induced microvascular damages in the adipose tissue. The significant alterations of HDL cholesterol, free fatty acids, CEL, PAS staining, TGF-*β* precursor, Masson Trichrome staining, Bcl-2/Bax ratio, and partially vWF were reverted by pyridoxamine. In accordance with previous reports from our laboratory, MG administration during 8 weeks did not have significant alterations in body weight, glycemia, and HbA1c [1, 7]. Once no alterations were found in the glucose metabolism, the lesions observed after MG treatment are only due to direct MG effects. However, MG administration increased systemic free fatty acids levels, suggesting adipose tissue dysfunction, due to a higher rate of lipolysis. These results are also in accordance with our previous observations after MG treatment during 14 weeks [1]. As demonstrated before in Zucker diabetic rats (ZDF) [13, 15], pyridoxamine effects led to lower systemic levels of free fatty acids. Our previous and present results show that MG treatment leads to decreased serum adiponectin levels, but these effects may be acquired during the time, as they are significant only after 14 weeks of MG administration [1, 7]. MG treatment during 8 weeks may have a lower impact on adiponectin levels, or the effects may have been lost because the rats were kept with water during 4 weeks after MG treatment.

CEL was determined as it is an important AGE formed directly from the MG [8]. Previously, correlation between CEL levels and MG administration was observed in the aorta of spontaneously hypertensive rats [16] and in retina of GLO1 transgenic rats [6]. As expected, MG administration during 8 weeks led to increased CEL levels and accumulation of PAS-positive material in adipose tissue, in accordance to previous reports [1, 17]. However, 8 weeks of MG treatment were not sufficient to induce major alterations in RAGE levels in adipose tissue, what is in accordance with previous data from our laboratory (unpublished data). However, that kind of effect was observed in aorta after 14 weeks of MG administration [7].

Previous *in vitro* studies from Onorato and colleagues showed that pyridoxamine inhibits AGEs formation as well as subsequent protein modifications, features commonly observed in type 2 diabetes [18]. Several authors described that pyridoxamine inhibits AGE formation through the reaction with and scavenging of dicarbonyl intermediates [11, 19]. The role of pyridoxamine in decreasing AGE formation in *in vitro *systems [18] and in different animal models, including STZ-induced diabetic rats and ZDF rats, was also observed [11]. In accordance, in our study the treatment with pyridoxamine during 4 weeks after MG administration caused a significant decrease of CEL levels and PAS-positive components in adipose tissue.

Previous studies showed that MG induces microvascular damages and diabetes-like complications. *In vivo *studies using Wistar rats showed an increase of TGF-*β* in glomerular, tubular, interstitial, and endothelial cells of the kidney [17]. *In vitro*, collagen glycation caused by MG stimulates the differentiation of human cardiac fibroblasts to myofibroblast, in a TGF-*β*-dependent manner. This process was described as critical for the development of fibrosis in diabetes [20]. As well, we reported that MG administration during 14 weeks also led to increased TGF-*β* expression, as well as increased Masson Trichrome staining [1]. In the present study, we show that even after 8 weeks of MG treatment a higher accumulation of Masson Trichrome components occurred. *In vitro* studies with pyridoxamine revealed that it protects from MG-induced integrin binding and cell adhesion, a major event of vessel thickening [10]. Here, we show that the treatment with pyridoxamine decreased the expression of TGF-*β* precursor and inhibited the accumulation of Masson Trichrome-positive material, suggesting a causal role between increased glycation markers and profibrotic responses. Colocalization of TGF-*β* and vWF suggests TGF-*β* expression to occur mostly in blood vessels regions. This demonstrates the presence of fibrosis in the microvasculature of adipose tissue, what may be an important factor for local microvascular lesions.

The ratio between Bcl-2 and Bax decreased after MG treatment, what is in accordance with several reports from our and other laboratories [1, 21, 22]. Our group demonstrated that decreased Bcl-2/Bax ratio is associated with increased endothelial cell death and higher vessel dysfunction in the retina [21]. Furthermore, we demonstrated that this event is also related with decreased blood supply to adipose tissue, what may lead to hypoxia, a major contributor to adipocyte dysfunction [1]. Pyridoxamine was observed to prevent cell death by apoptosis in the human lens epithelial cell line HLE-B3 and in the cataractous lenses of ZDF rats, through the inhibition of argpyrimidine formation [5, 23]. In B6 *db/db* mice, a model of type 2 diabetes, pyridoxamine decreased the progression of established diabetic nephropathy [14].

As mentioned, we previously reported that MG administration during 14 weeks causes a decrease of blood supply to the adipose tissue, mainly through increased endothelial cell death and impaired angiogenesis. In this study, we show that these effects were similar after 8 weeks, although more modest. Despite pyridoxamine was effective in the reduction of most of the lesion markers, it was not able to significantly raise adipose vWF levels in relation to MG-treated rats. However, vWF levels were no longer decreased in relation to control rats, suggesting a partial effect.

## 5. Conclusions

We show that reducing adipose tissue glycation and fibrosis using pyridoxamine may be a useful strategy to improve adipose tissue microvascular lesions. This may contribute to prevent adipose tissue dysfunction during type 2 diabetes progression.

## Figures and Tables

**Table 1:** Body weight, glycemia, fasting and 2 hours after intraperitoneal glucose injection (1.8 g/kg), glycated hemoglobin, serum triglycerides, and total and HDL cholesterol.

Parameter	C	MG	Pyr
Body weight (g)	430 ± 17	440 ± 18	433 ± 12
Fasting glycemia (mg/dL)	63.3 ± 1	62.9 ± 1.5	64.3 ± 2
Glycemia at 2 h (mg/dL)	92.0 ± 6.1	91.3 ± 1.8	92.5 ± 3.1
HbA1c (%)	3.6 ± 0.1	3.6 ± 0.0	3.7 ± 0.1
Triglycerides (mg/dL)	95.8 ± 9.1	78.6 ± 5.6	98 ± 10.8
Total cholesterol (mg/dL)	92.5 ± 5.8	84.7 ± 4.7	91.3 ± 4.5
HDL cholesterol (mg/dL)	55.5 ± 1.2	47.3 ± 2.0*	54.8 ± 1.6^#^

C: Control Wistar rats; MG: Wistar rats with methylglyoxal administration; Pyr: Wistar rats with methylglyoxal administration and pyridoxamine treatment. Data is presented as mean ± SEM, *n* = 6 in each group. *Different from C; ^#^different from MG. 1 symbol *P* < 0.05.

## Abbreviations

W: Wistar rats
AGEs: Advanced glycation end products
CEL: N-epsilon-(carboxyethyl)lysine
MG: Methylglyoxal
PAS: Periodic acid of Schiff
RAGE: Advanced glycation end-product receptor
TGF: Transforming growth factor
vWF: Von Willebrand factor.
